# Supplementary material for: Health, environmental, and animal rights motives for vegetarian eating
Source: PLoS One. 2020 Apr 2;15(4):e0230609. doi: 10.1371/journal.pone.0230609 (PMC7117663; doi:10.1371/journal.pone.0230609)
Supplement: Attachment — Submitted filename: review PLOSONE Health....docx [file pone.0230609.s001.docx]

This is an interesting and well-conceived/executed manuscript with the relevant aim of developing a questionnaire measure (Vegetarian Eating Motives Inventory) to assess the importance attached to (often cited) reasons for choosing a vegetarian or meat reducing diet. The design and the statistics/analysis is in general very high level. Although the paper primarily is a developmental one, there are some interesting results as well. The authors also have a number of points about the ramifications in a general population (where I think they should be a bit more cautious, cf point 10 below).

I have a number of suggestions.

1) You could specify more concretely in the abstract which population the Inventory is for. Only for vegetarians and vegans? Only for meat reducers or the general meat consuming population (which will make the use of the measure more clear for future interested researchers). I liked the paragraph in the Discussion (p 15) which perhaps could be used in a shorter format in the abstract? (“This measure has considerable promise for future research on the motivations for plant-based eating. Moreover, although our goal was to develop the VEMI to assess the potential motives of non-vegetarians in a general population, it can be easily adapted for research among vegans, vegetarians, flexitarians, reducetarians, and other groups.” )

2) Related to point 1: could be pointed out more clearly that, since the inventory exhibits invariance, it is likely to function well throughout Western societies (however, see point 6)

3) It is fine that it is stated that the inventory examines three most dominant reason in Western societies. You could mention already in the Intro that you exclude non-religious motivations that also exists in Western societies (e.g. regligous, hindus).

4) It is know from studies that some vegetarians/vegans develop meat disgust/distaste over time (see for instance your own ref 63). At some point this becomes an important motivation. You should probably mention that this was excluded from the Inventory in the *Limitations and Future Directions* section. It makes perfectly good sense to me that you excluded it, as you were interested in non-vegetarian converters (that arguably have not developed disgust).

5) For all samples, it should described whether there were exclusion criteria in general and owning to diet specifically (could any type of diet (meat eating status) participate?). The geographical and/or national composition of all samples should be specified directly in the Methods section (can only see that Sample 3 is Dutch). Also, considering the nature of your research endeavor, it would also be a good idea to report in the Method section how many vegetarians, vegans, meat reducers there were in each sample.

6) Your claim of invariance could be strengthened and improved if further multigroup measurement invariance assessments were carried out. You could look at gender (in all samples), age (in sample 2 and 3 where there is much age variation). For sample 1 you could also determine “broader cultural” invariance. Thus, you have students with nonwestern heritage (sample 1: 485 Asians). The analysis may not be strictly necessary, but it seems just as important to look at invariance related to actual empirical differences and not only sample differences.

7) I like the introduction and motivation for developing the inventory very much – it is clear and to the point. Many relevant former measures are discussed.

On page 3: you write “ a more ethical approach to inter-species relationships”. What is meant with a more ethical approach? This appears too normatively motivated. An anthropocentric ethical approach is just as ethical as an animal rights approach. Just another kind of ethics. Please reformulate to the specific ethics approaches (animal rights or whatever) that the references suggest.

You could say that you also collected a sample 4 in the latter paragraph before the method section (p7), since you also reported about the three other samples in the Intro.

8) Tables:

Table 5: please add notes to the tables specifying a) what the numbers in the table indicate (betas?), b) for each row whether it was just one or all of the scales that had to be significant for the association to be reported in the table, and 3) type of analysis (distributional-wise (logit, linear or whatever)) the table reports from.

Table 6: please add notes to the tables specifying a) what type of correlational test that is reported (pearson, spearman, etc).

The table 7 note: “association predicted in LISS” should be phrased to “prediction of associations to be tested in LISS” to avoid confusion.

9) In the limitation section (p 20) you mention that a useful extension could be to study motivations outside of peoples’ conscious awareness. I am not sure they are motivations anymore when they move away from awareness, but maybe implicit attitudes. This has been studied for meat eaters actually. Anyway, another way to look at it, and that you might consider including as a future research avenue, could be to prompt people for what is most important (best-worse scaling)

10) The design of the correlational analyses:

My primary concern of the study relates to the methodology of the correlational analyses. You

i) use two convenience samples (from different populations) to determine the *Correlates that are looked for in the Plant-Based Eating Motives* in Sample 3 (LISS).

ii) Following exclusions on basis of i) and what could be replicated in the LISS sample, you conclude quite much about relevant criterions (p17 “The only criterion reliably related to environmental motives was participation in an environmental rights organization. No criteria were reliably related to animal rights motives across all three samples. These circumscribed findings for the environment and animal rights scales surprised us given the large number of correlates we examined” and later that (p 21) “health motives have the widest array of correlates, which generally involve agentic and communal values.”).

This makes me wonder whether you may be relying too much on the two convenience samples to exclude possible relevant findings (and ACTUAL population differences) from sample 3?

In general, I think that the idea about determining “relevant/valid” criterion variables through three samples need more clarification. There are a number of questions that I am left with:

A) First of all, why did you choose to do three samples to determine the final (potential) relevant criterions? Why not five? Or ten?

B) Is is valid to study this with varying populations (students, Americans (I suppose, in sample 2), and people from the Netherlands). Any of the correlations (or non-assiciations) that could not be replicated in sample 2 and 3 could be a consequence of actual associational differences in the populations compared to the baseline in sample 1 (sample 1 consists of soon to be highly educated and young individuals – and many women that also could have a big impact).

In short, I do not think that the preregistration procedure related to the Sample 3 analysis is particular useful considering the aim of detecting relevant associations in a general population because of the aforementioned points A and B. I realize that you have put much effort into this part of the analysis and I am not asking you to remove this (you may also disagree with my remarks). But you could at least consider mentioning some of these limitations in your discussion (and rebut them if you disagree). But your claims about the general population (returning to point ii above) also rest on this procedure and you should as a minimum state in the Conclusion that the population results (your array of correlates) may be incomplete because of the measure developmental character of the paper and that future studies should examine this further.

11) Following up on the former: you mention “large effects distinguished the 97 vegans across all three samples from non-vegan respondents for the health (d = .51), environment (d = 1.29), and animal rights (d = .97). But you report no significant correlations with vegans in sample 3 - LISS data Table 7. Because there was a little correlation in sample 1 (r 0.12). Could this be because of too few vegans in sample 1 so the small correlation is by chance? Related to this: I cannot understand that you mention that you excluded correlational candidate variables in sample 1 and 2 if “less than 50 participants responding either “no” or “yes” – p 13.) But if you had an inclusion criteria of 50 in the two first samples and there only are in total 97 vegans in all three samples I cannot see how vegans should be included in the pool of correlational variable in the first place.

Could you please explain (I may be overseeing something)?

12) It seems that you excluded “All zero” from the subsequent follow-up correlation identification. Why? It seems just as important to show that non-associations replicate (this is a case of discriminant validity). Please explain why you were conceptually interested only in replications of significant associations.
